# Advanced Bio-Based Smart Materials for Food Packaging: Applications, Safety, and Sustainability

**DOI:** 10.3390/foods14203462

**Published:** 2025-10-10

**Authors:** Ioannis Konstantinos Karabagias

**Affiliations:** Department of Food Science and Technology, School of Agricultural Sciences, University of Patras, G. Seferi 2, 30100 Agrinio, Greece; ikaraba@upatras.gr; Tel.: +30-697-828-6866

## 1. Introduction

The greatest issues facing humanity today are food security, safety, and waste management, as well as the pernicious impacts of environmental climate change. Thus, the development of intelligent technologies pertaining to the wholesomeness of food products or their waste is essential. The petroleum-based plastics used in food packaging materials, which have recently raised numerous concerns regarding environmental pollution and consumer health, may be replaced by these technologies in the future, which could potentially lead to a complete recharacterization of the quality of the product from production to packaging and distribution [1].

Advanced packaging materials better qualities and sensors for that can track the condition of food in storage are just two examples of the “smart” features that sophisticated technology and materials science have introduced to food packaging. Recently, smart packaging has been used to extend the shelf life of foods. This includes time–temperature indicators, modified atmosphere packaging sensors for monitoring CO_2_ and O_2_, total volatile base nitrogen sensors for detecting food deterioration, fruit ripeness indicators, pathogen sensors [2], and protocols for food tracking and authentication that have not yet been studied in detail.

Advanced bio-based materials in the context of packaging can be used in biodegradable matrices as well as synthetic polymers. Smart packaging prolongs food freshness and offers more comprehensive food chemical monitoring. Furthermore, compared to traditional plastic packaging, the environmental impact of biodegradable films or coatings is minimal [3].

The main categories of smart packaging are (i) intelligent packaging and (ii) active packaging. Biosensors, gas sensors, microbial indicators related to freshness, and time–temperature indicators are mainly used in intelligent packaging. Active packaging, on the other hand, includes primarily antibacterial and antioxidant compounds, as well as gas, moisture, and odor absorbers [1]. These substances give films or coatings their bioactive functionality. They are generally chitosan, cellulose, essential oils, organic acids, fungicides, bacteriocins, antibiotics, enzymes, alcohols, whey protein, and lipids that have been incorporated into the packaging material [3].

Recent trends in advanced bio-based materials used in food packaging also include the usage of nanocomposites carrying antimicrobials, which are a promising tool for decreasing the risk of pathogenic bacteria growth and prolonging the shelf life of perishable foods, demonstrating advantages over the standard packaging of food products [3,4]. However, a gap remains in the use of polymer nanocomposite films and coatings, given the safety concerns that may arise [5].

Considering the above, this Special Issue aimed to collate research on the most recent and advanced bio-based smart materials used in food packaging, including their applications, safety issues, and sustainability.

## 2. An Overview of Published Articles

**Figure 1:**
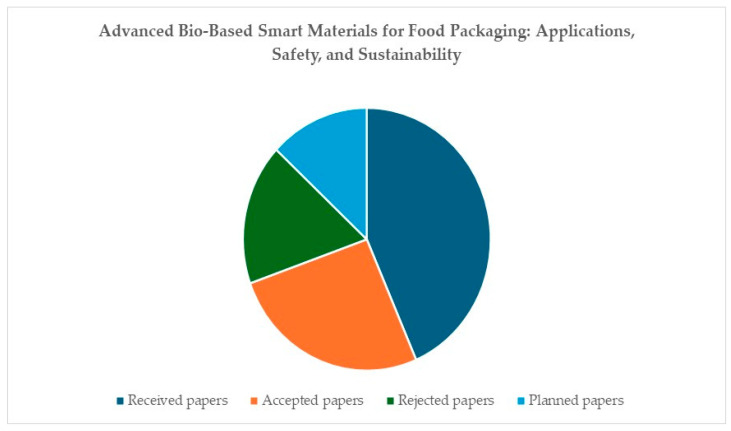
Special Issue metrics.

The current Special Issue received ten submissions, among which six were published and four were rejected. Three additional submissions were planned papers that were not submitted until the end date of this Special Issue (Figure 1).

The published articles focused on the most recent trends in smart materials and bioactive packaging. In particular, Safakas et al. [6] investigated the fabrication of packaging materials with antibacterial and antioxidant properties to prolong the shelf life of baguette bread. Using X-ray diffraction, tensile testing, oxygen permeability measurements, and antioxidant assays, the authors created and characterized low-density polyethylene (LDPE) and poly(ethylene-co-vinyl acetate) (EVA) films that incorporated organically modified montmorillonite (OMt) nanocarriers loaded with components of carvacrol (C) and thymol (T) essential oils. Both the LDPE/EVA/OMtC and LDPE/EVA/OMtT films demonstrated significant antioxidant activity and enhanced mechanical strength. Furthermore, films made with an 80/10/10 component weight ratio of LDPE/EVA/OMtC demonstrated modified barrier qualities and prevented fungal development on baguette bread for as long as 60 days.

Liu et al. [7] addressed the problem of biopolymer–anthocyanin intelligent packaging films with high hydrophilicity, which severely restricts their use in high-humidity settings. In order to overcome the hydrophilicity of purple-sweet-potato-based intelligent packaging films, the authors created a surface hydrophobization process that involved coating with stearic acid and heat pressing at temperatures of 100 and 150 °C. The films’ structural features, physical attributes, and color changeability were the parameters that were measured. The stearic-acid-coated films showed reduced transparency, mechanical characteristics, moisture content, surface wettability, anthocyanin leaching capability, and color changeability when compared to the untreated purple sweet potato films. The heat-pressed films outperformed the stearic acid-coated films in terms of transparency, mechanical properties, and water vapor blocking capacity. The scientists also noted that the heat pressing temperature had an impact on the color and color changeability of the heat-pressed films. In contrast to the films heat-pressed at 150 °C, which took on a brown hue and lost color changeability, the films heat-pressed at 100 °C displayed a vibrant purple hue and increased color changeability. Based on these results, the scientists concluded that heat pressing and stearic acid coating comprise a well-featured procedure to create surface-hydrophobized intelligent packaging films.

Ren et al. [8] focused on the creation of pH-sensitive materials for seafood (hairtail) freshness monitoring in real time. Using the solution casting approach, the authors added purple cabbage anthocyanin (PCA) in varying percentages (2.5%, 5.0%, 7.5%, and 10%, *w*/*w)* to a κ-carrageenan/carboxymethyl cellulose (CA/CMC) matrix to create pH-responsive indicator films. According to the authors, the produced films have a high pH sensitivity (pH range: 2.0–11.0). The researchers also showed that the PCA incorporation decreased water vapor transfer while improving film opacity, antioxidant qualities, and crystallinity. Rougher morphology and decreased tensile strength were the results of the greater PCA content, although elongation at break was improved. After ten days in the dark and during cold storage at 4 °C, the color difference was not discernible, demonstrating the indicator films’ good environmental resilience. The CA/CMC/PCA (10% *w*/*w*) film had the most pronounced pH-responsive color changes, transitioning from purple to green in relation to hairtail freshness.

Given that the quality of winter jujube fruit declines after refrigeration, Hei et al. [9] combined low-temperature storage (at 0 ± 1 °C, relative humidity: 90 ± 5%) with melatonin in varying concentrations (100, 150, and 200 µmol/L) to increase the shelf life of harvested winter jujube fruit, a well-known perishable fruit. The fruit’s firmness, redness, weight loss, total soluble solids, titratable acidity, and the amounts of total phenols, flavonoids, glutathione, and ascorbic acid were among the quality indicators that the authors measured every 15 days. The findings demonstrated that varying melatonin doses might preserve the firmness of the fruit, delay its redness, and prevent weight loss, total soluble solids, titratable acidity, and the levels of ascorbic acid, glutathione, flavonoids, and total phenols. In addition, low-temperature storage and melatonin increased the activities of antioxidant enzymes (superoxide dismutase, catalase, ascorbate peroxidase, glutathione reductase, and peroxidase) and reduced the rise in the relative conductivity, malondialdehyde content, and hydrogen peroxide content of jujube fruits. The authors found that 200 µmol/L melatonin and low-temperature storage had the greatest effects on the fruit’s ability to maintain its quality while also enhancing its antioxidant capability.

Da Silva Simões et al. [10] examined the effectiveness of a novel edible coating made of alginate and fungal chitosan functionalized with *Lacticaseibacillus casei* (LC03) in both free and microencapsulated forms to improve the nutritional value and shelf life of strawberries. *L. casei* cells were successfully encapsulated in alginate microparticles and then covered with chitosan, according to the authors, which improved the cells’ survival, protection, and encapsulation efficiency. By preserving pH, titratable acidity, moisture, and microbiological quality, the edible coating containing *L. casei* microencapsulated in alginate and covered with fungal chitosan greatly enhanced strawberry preservation. Furthermore, strawberries stored in a refrigerator at 4 ± 1 °C and 85–90% relative humidity for 12 days showed a delayed decrease in total phenolic compounds. While coatings containing fungal chitosan showed less weight loss, those containing free *L. casei* showed a small reduction in color. Overall, the alginate–chitosan coating and microencapsulated *L. casei* demonstrated their ability to preserve the nutritional value, safety, and quality of strawberries, while they were stored in a refrigerator, underscoring their potential for the development of environmentally friendly and useful packaging.

The study of Panou et al. [11] included 186 references in a thorough review article about the use of smart packaging systems, or sophisticated packaging systems, to preserve perishable goods like fruits (tree fruits, berries, stone fruits, and aggregate accessory fruits) during storage and transit. The writers made an effort to draw attention to the distinctions between active and intelligent packaging. To preserve the quality of plant-based foods and increase their shelf life, they also highlighted the distinctions between biosensors and gas sensors; microbial, freshness, and time–temperature indicators (intelligent packaging); moisture, odor, and gas absorbers; and between antioxidant and antimicrobial agents (active packaging).

## 3. Conclusions and Future Perspectives

Intelligent and active packaging (smart packaging) protects food from the effects of numerous physical, chemical, and biological hazards. The advantages concerning safety, logistics, and marketing show that smart packaging could be adopted by the food industry in the near future [2]. However, there is still much work to do to fill the gap regarding the compatibility of each package with food. Therefore, there is also a need to produce authentic packages used for the maintenance of the quality of each specific food. Additionally, food tracking systems should be developed to monitor and address any possible problems related to the distribution and application of advanced bio-based materials used in food packaging, along with migration testing of the incorporated bioactive components into foods, especially when nanomaterials are applied. To overcome these concerns, there is a great need for beneficial cooperation between research institutes and the food industry at all levels of preparation, application, and distribution of these films or coatings.

## Data Availability

All research data are found in the Special Issue link: https://www.mdpi.com/journal/foods/special_issues/B8XPGBLSSJ, accessed on 23 September 2025.
